# *In vivo *and *in vitro e*valuation of an *Acetobacter xylinum *synthesized microbial cellulose membrane intended for guided tissue repair

**DOI:** 10.1186/1751-0147-51-12

**Published:** 2009-03-24

**Authors:** Péricles Nóbrega Mendes, Sheila Canevese Rahal, Oduvaldo Câmara Marques Pereira-Junior, Viciany Erique Fabris, Sara Lais Rahal Lenharo, João Ferreira de Lima-Neto, Fernanda da Cruz Landim-Alvarenga

**Affiliations:** 1 Department of Veterinary Surgery and Anesthesiology, School of Veterinary Medicine and Animal Science, São Paulo State University (Unesp), Botucatu, SP, Brazil; 2 Department of Pathology, Botucatu Medical School, Unesp, Botucatu, SP, Brazil; 3 Department of Federal Police, Brasília, Distrito Federal, Brazil

## Abstract

**Background:**

Barrier materials as cellulose membranes are used for guided tissue repair. However, it is essential that the surrounding tissues accept the device. The present study histologically evaluated tissue reaction to a microbial cellulose membrane after subcutaneous implantation in mice. Furthermore, the interaction between mesenchymal stem cells and the biomaterial was studied *in vitro *to evaluate its ability to act as cellular scaffold for tissue engineering.

**Methods:**

Twenty-five Swiss Albino mice were used. A 10 × 10 mm cellulose membrane obtained through biosynthesis using *Acetobacter xylinum *bacteria was implanted into the lumbar subcutaneous tissue of each mouse. The mice were euthanatized at seven, 15, 30, 60, and 90 days, and the membrane and surrounding tissues were collected and examined by histology.

**Results:**

A mild inflammatory response without foreign body reaction was observed until 30 days post-surgery around the implanted membrane. Polarized microscopy revealed that the membrane remained intact at all evaluation points. Scanning electron microscopy of the cellulose membrane surface showed absence of pores. The *in vitro *evaluation of the interaction between cells and biomaterial was performed through viability staining analysis of the cells over the biomaterial, which showed that 95% of the mesenchymal stem cells aggregating to the cellulose membrane were alive and that 5% were necrotic. Scanning electron microscopy showed mesenchymal stem cells with normal morphology and attached to the cellulose membrane surface.

**Conclusion:**

The microbial cellulose membrane evaluated was found to be nonresorbable, induced a mild inflammatory response and may prove useful as a scaffold for mesenchymal stem cells.

## Background

The composition and structure of a membrane are important factors in determining its medical applications [1,2]. Membranes constructed of synthetic or semisynthetic materials (polytetrafluoroethylene, expanded polytetrafluoroethylene, polylactic acid, copolymer of polylactic acid and polyglycolic acid, cellulose acetate, and others) or of natural origin (type I bovine collagen, porcine type I collagen, bovine type I atecollagen, and others) have been developed and tested, with some of them showing promising results as barrier material [3-7].

Barrier materials have been used to promote guided bone regeneration or guided tissue regeneration in maxillofacial bone defects, cranial defects, and periodontal bone defects [5-8]. The same biological concept has been used in the treatment of segmental defects in long bones [3,9,10]. Both resorbable and nonresorbable membranes have been developed, each of which with advantages and disadvantages. Within dentistry, the problems associated with nonresorbable barrier materials include requirement of a second surgical procedure for removal of the membrane besides gingival recession and membrane exposure [11]. On the other hand, the resorbable membranes should preferably be resorbed in a time period that is predictable and compatible with the bone regeneration and the degradation should not interfere with bone regeneration [12].

Membranes composed of bacterial cellulose produced by *Acetobacter *species have been tested clinically and experimentally for different applications, such as wound dressing material, duraplasty, nerve anastomosis, artificial blood vessels or barrier to bone defects [2,13-19]. Differences in the manufacturing according to initial concentrations of carbon sources, surface/volume ratios, strain of *Acetobacter *and extended times of fermentation interfere in the final product obtained [2,19]. In addition, the choice of a particular cellulose structure will depend on the clinical application [2].

The multipotent mesenchymal stem cells are becoming a subject of increasing interest because of their potential in tissue engineering applications [20]. They can be obtained from adult bone marrow and are capable to differentiate into other phenotypes including the cells of the bone, cartilage, tendons, ligaments, fat, and other connective tissues [21]. According to tissue engineering concepts, it is possible to regenerate various tissues using living cells and an appropriate scaffold [22].

Thus, the aim of the present study was to histologically evaluate tissue reaction to a cellulose membrane obtained through biosynthesis using *Acetobacter xylinum *and its *in vitro *ability as scaffold to mesenchymal stem cells.

## Materials and methods

### Biomaterial

The membrane evaluated in this study was provided by the manufacturer (Bionext; São Paulo, Brazil). It is approved for medical use by the Brazilian Health Agency – ANVISA (register number 80255120001). The membrane is semi-transparent, flexible, hydrophilic, selective permeable, pH 6.0–7.0, 0.05 mm thick, and gamma radiation-sterilized.

### Evaluation of membrane surface

For the evaluation of the biomaterial's surface, membrane pieces of 1 cm^2 ^were submitted to scanning electron microscopy in a Quanta 200 3D Scanning Electron Microscope (Fei Company, Hillsboro, USA). No previous preparation of the samples was needed.

### Tissue reaction evaluation

Twenty-five male Swiss Albino mice, approximately 1 month old and weighing 25 g were used. The animals were randomly divided into five groups according to postoperative observation points (G1 = 7 days, G2 = 15 days, G3 = 30 days, G4 = 60 days, G5 = 90 days). Each group of five mice was housed in a polyethylene cage (30 × 20 × 13 cm) with a stainless steel top. Commercial rat chow diet and water were provided *ad libitum*. Guidelines for the care and use of laboratory animals were followed and the study was approved by the Ethics Committee at the School of Veterinary Medicine and Animal Science, São Paulo State University.

Before surgery, anesthesia was induced by intramuscular injection of a combination of xylazine 2%, 10 mg/kg (Bayer S.A., São Paulo, SP, Brazil) and ketamine 5%, 150 mg/kg (Vetbrands Brasil Ltda., Paulínia, SP, Brazil). Each mouse was positioned in ventral recumbency and the lumbar area was prepared aseptically for surgery. A skin incision (1 cm) was made dorsolaterally in the right flank area. A piece of membrane (10 × 10 mm) was placed below the dorsal midline after blunt dissection through the subcutaneous tissues. Skin incision was closed using simple interrupted sutures of monofilament nylon 4-0. Buprenorphine (Schering Plough, Rio de Janeiro, RJ, Brazil) was administered intramuscularly immediately after surgical procedure (25 mg/kg).

Groups of five mice were euthanized at seven, 15, 30, 60, and 90 days post-surgery by intraperitoneal administration of an overdose of sodium pentobarbital. The membrane and surrounding tissues were collected and stored in 10% phosphate buffered formalin. After fixation, specimens were washed in tap water for 5 h, dehydrated in ethanol, cleared with xylene and embedded in paraffin. Histological sections of 5 μm thickness were stained with hematoxylin and eosin. The specimens were evaluated under polarized and light microscopy. Descriptive analysis of inflammatory infiltrate, vascular density and fibrosis was done. Semi-quantitative tissue analysis using previously established scores was performed as follows: absent (0), mild (1), intense (2), and severe (3).

The data were submitted to statistical analyses. Analysis of variance followed by the Tukey-Kramer Multiple Comparisons Test was used to evaluate the five different time points using the GraphPad InStat software. Differences were considered statistically significant at *P *< 0.05.

### Biomaterial in cell culture

Canine bone marrow (5 ml obtained from the humerus) was collected for cell culture and centrifuged at 300 g for 10 min to remove serum and fat. The cell rich sediment was then diluted at the proportion of 1/1 with Dulbecco's Modified Eagle Medium (DMEM) high glucose with L-glutamin (GIBCO BRL; Grand Island, USA). Four ml were transferred to a tube containing 4 ml of Ficoll-Paque (1.077 g/ml) for density gradient centrifugation at 300 g for 40 min. After this, the mononuclear cell ring was collected and washed with DMEM twice. The cells were then diluted in 1 ml DMEM with 20% fetal calf serum (FCS) and transferred to culture bottles of 25 cm^2 ^with 5 ml of DMEM (with L-glutamine), FCS, penicillin and streptomycin. Once the cells achieved 80% of sub-confluence at 15 days of culture, they were re-suspended to a concentration of 2 × 10^7 ^cells/ml.

To confirm the mesenchymal stem cell lineage, CD34 and CD44 specific surface antibodies (AbD Serotec, Oxford, UK) were used to mark mesenchymal cells. The cell populations isolated on primary culture were prepared according to the antibodies manufacturer protocols. The CD34 antibody FITC (Fluorescein Isothiocyanate) conjugated was negative at direct immunofluorescence staining for flow cytometry. The CD44 antibody associated to RPE secondary antibody (R. Phycoerythrin) was positive at indirect immunofluorescence staining for flow cytometry. The tests were performed by a flow cytometer (FACS Calibur – BD).

The microbial cellulose membrane was cut to fit into a 6-well plate. The membrane was damped with FCS and each well was filled with 5 ml of a medium containing DMEM, 20% FCS, penicillin, streptomycin and amphotericin B before mesenchymal stem cells were added. The stem cells were placed over the cellulose membrane at a concentration of 1–2 × 10^6 ^cells/ml. The cells were incubated at 37.5°C in a 5% CO_2 _atmosphere. The cell growth was followed over 10 days and the cells were subsequently submitted to cell viability staining with Hoescht 33342 (Sigma Chemical Co, St. Louis, USA) and Propidium Iodide (Sigma Chemical Co). Scanning electron microscopy was used to evaluate the cell attachment and growth. The membrane samples associated to the mesenchymal stem cells were removed from the 6-well plate and directly assessed in a Quanta 200 3D Scanning Electron Microscope (Fei Company, Hillsboro, USA). No previous preparation of the samples was needed.

## Results

**Figure 1:**
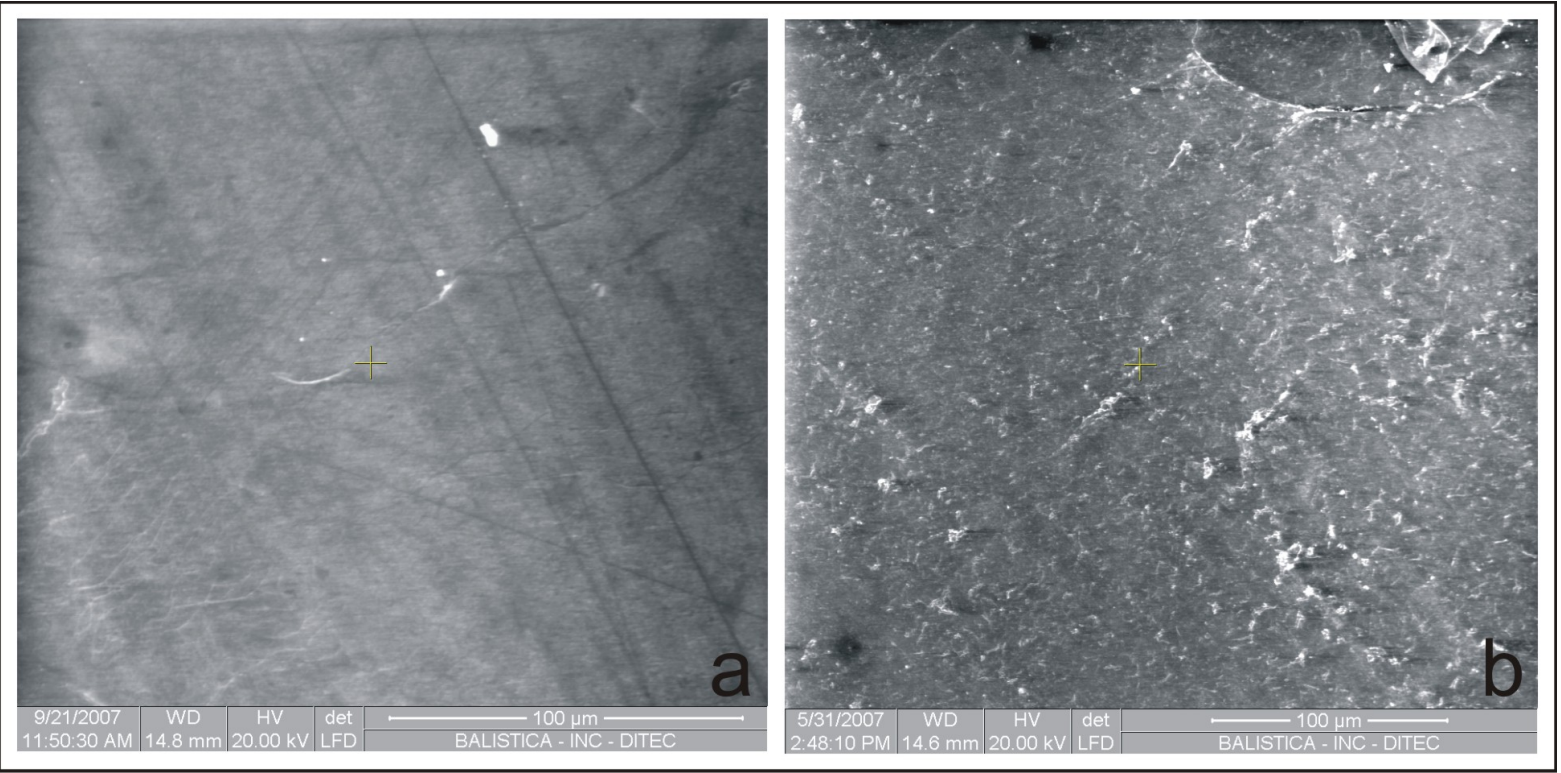
**Scanning electron microscopy of both sides of a microbial cellulose membrane**. One side of the membrane is completely smooth (a), and the other side is distinctly rough (b).

Scanning electron microscopy of the microbial cellulose membrane showed absence of pores throughout its surface. One side of the membrane was completely smooth (Fig. 1a), while the other side was distinctly rough (Fig. 1b).

**Figure 2:**
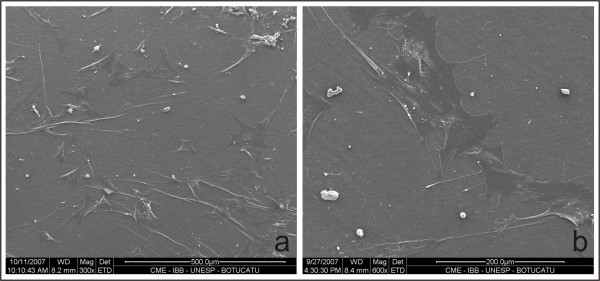
**Scanning electron microscopy of a microbial cellulose membrane after 10 days in cell culture**. Observe the mesenchymal stem cells with normal morphology and attached to the membrane surface. (×1200).

The viability staining analysis of the cells over the biomaterial showed that 95% of the mesenchymal stem cells aggregating to the cellulose membrane were alive while 5% were necrotic. Scanning electron microscopy of the mesenchymal stem cells showed normal morphology and attachment to the cellulose membrane surface (Fig. 2a and 2b).

**Figure 3:**
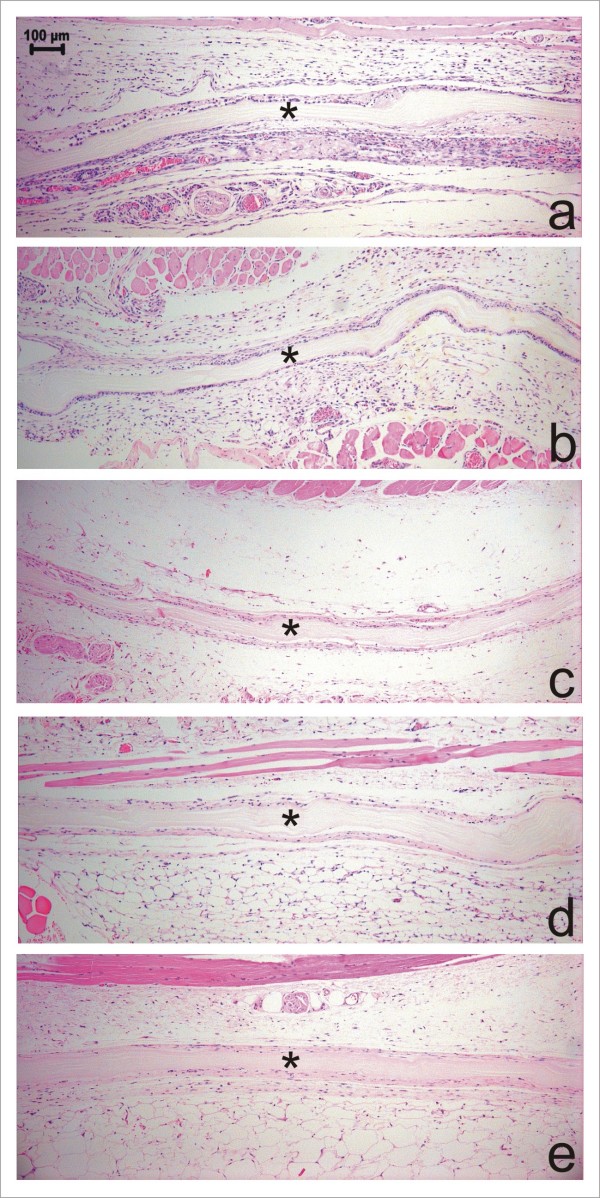
**Histomorphology of a microbial cellulose membrane implanted subcutaneously in mice and surrounding tissue reaction 7(a), 15(b), 30(c), 60(d) and 90(e) days postoperatively**. Observe the presence of the intact membrane (*) surrounded by immature granulation tissue and newly formed vessels and capillaries (a). At 15 days post-surgery, a reduction in inflammatory infiltrate, especially of lymphocytes, is observed (b). At 30 days postoperatively observe the collagen fibers commencing orientation parallel to the implant's surface (c). No inflammatory infiltrate is observed and the connective tissue surrounding the membrane is mature at 60 (d) and 90 (e) days post-surgery. (HE, Obj. ×10).

**Figure 4:**
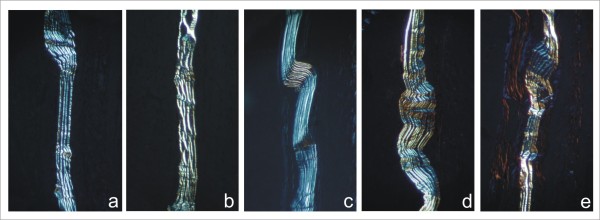
**Polarized microscopy showing the structural organization of the cellulose membrane**. There is no evidence of structural organization alteration at 7 (a), 15 (b), 30 (c), 60 (d) and 90 (d) days postoperatively. (Obj. ×10).

Descriptive analysis of the histological sections by light microscopy on day seven post surgery demonstrated an intact membrane surrounded by a mild inflammatory infiltrate of mainly polymorphonuclear cells and lymphocytes (Fig. 3a). Immature granulation tissue was evinced by intense presence of newly formed vessels and capillaries close to the membrane and mononuclear cells. Examination of mice 15 days post-surgery showed a reduced inflammatory infiltrate, especially due to lower numbers of lymphocytes (Fig. 3b). Granulation tissue appeared similar to that observed at day 7 post surgery. The membrane showed no signs of resorption. No polymorphonuclear cells and only a few lymphocytes were observed at 30 days post-surgery. There was a reduced number of newly formed vessels and collagen fibers began to be oriented parallel to the implant's surface that was apparently intact (Fig. 3c). At 60 and 90 days post-surgery, no inflammatory infiltrate was observed. Angiogenesis was markedly reduced and the connective tissue surrounding the membrane was mature. The membrane was still present with no signs of resorption (Figs 3d and 3e). Foreign body reaction or connective tissue cells penetrating the membrane were not observed at any time point throughout the study period. Polarized microscopy revealed that the membrane remained intact at all evaluation points (Fig. 4). Table 1 summarizes the mean score values (semi-quantitative analysis) regarding the intensity of inflammatory infiltrate, polymorphonuclear cells, multinucleated giant cells, lymphocytes, angiogenesis, and fibrosis. No significant differences were observed among the time points regarding presence of polymorphonuclear cells, multinucleated giant cells, and lymphocytes (*P *> 0.05). Angiogenesis and fibrosis decreased throughout the evaluation periods (*P *< 0.05).

**Table 1:** Scores^1^attributed to the level of infiltration with polymorphonuclear cells (PMNs), multinucleated giant cells (MGCs), and lymphocytes, and development of angiogenesis and fibrosis at seven, 15, 30, 60 and 90 days post-operatively.

	**Time points of evaluation (days)**				
	
**Cells/event**	**7**	**15**	**30**	**60**	**90**
**PMNs**	0.5^2 ^(0/1)^3,a^	0.5 (0/2)^a^	0 (0/0)^a^	0 (0/0)^a^	0 (0/0)^a^
**MGCs**	0 (0/0)^a^	0 (0/0)^a^	0 (0/0)^a^	0 (0/0)^a^	0 (0/0)^a^
**Lymphocytes**	1 (0/2)^a^	0 (0/0)^a^	0.2 (0/1)^a^	0 (0/0)^a^	0 (0/0)^a^
**Angiogenesis**	2 (1/3)^a^	2 (1/3)^ab^	1 (1/2)^ab^	1 (1/1)^ab^	1 (0/1)^b^
**Fibrosis**	1.5 (1/3)^a^	1.5 (1/3)^a^	1 (1/1)^ab^	1 (0/1)^ab^	0 (0/1)^b^

^1 ^Scores: absent (0), mild (1), intense (2), and severe (3);

^2^Mean score of analysis;

^3^The number inside parenthesis represents the minimal and the maximal score observed in the event;

Values followed by different letters (a or b) on horizontal were significantly different (*P *< 0.05)

## Discussion

The chemical composition of a membrane intended for guided tissue repair determines the type, duration and degree of inflammatory and immune response, means of disintegration and its longevity in the host tissue [1]. The inflammatory response may delay the healing process [23]. In the present study a low inflammatory response to the implanted membrane was seen at seven, 15, and 30 days post-surgery with absence of foreign body reaction at any time, suggesting that the microbial cellulose membrane was well tolerated by the organism. Low cellular reaction was also found in a duraplasty study in dogs [14], and no gross or histological signs of inflammation including giant cell reaction were observed when pieces of bacterial cellulose membranes were implanted subcutaneously in rats for one to 12 weeks [24]. In addition, the infection rate was decreased in humans that received cellulose membrane as wound and burn dressings [13]. The absence of multinucleated giant cells suggests absence of foreign body reaction [1,25]. Absence of foreign body reaction is important as such reactions may demand additional surgery for removal of the device [1].

In general, chemically nonreactive smooth-surfaced implants are surrounded by fibroblasts and collagen oriented parallel to the implant's surface within 2 weeks of implantation [1,25]. Connective tissue surrounding but not penetrating the membrane was observed as early as 7 days postoperatively in the present study. Later on, especially 30 days after surgery, improved collagen deposition was seen. Similar enveloping of a cellulose membrane by connective tissue has been observed in association with duraplasty [14]. Other authors have noticed that fibroblasts are able to penetrate the more porous bottom side of a cellulose membrane implanted into rats [24].

In the present study no signs of membrane structural changes or membrane absorption were detected by light and polarized light microscopy. In a clinical study comparing cellulose membrane and expanded polytetrafluoroethylene as barrier membrane in the treatment of class II furcation in human patients, both materials were removed 4 weeks after placement [15]. On the other hand, in a duraplasty study in dogs the membrane was invaded by connective tissue and membrane filaments had loosened and separated from each other, resulting in its partial disappearance when evaluated 270 days postoperatively [14]. Differences in production techniques probably influence the results [2,19]. However, since the membrane used in the present experiment seems nonresorbable, problems associated with membrane durability may emerge [11].

Guided bone regeneration presents some requirements such as prevention of bacterial infection, maintenance of space beneath the barrier membrane, and separation of osteogenic cells from the competing nonosteogenic cells [1,26]. Since the tested cellulose membrane was flexible, especially when wet, it is probably unable to prevent soft tissue collapse into a bony defect, which would necessitate the placement of a bone graft or biomaterial together with the membrane as a space-holder [1,3]. This scenario was probably one of the factors that influenced the incomplete bone regeneration in circular defects performed on rabbit tibia [18].

Some authors describe the microbial cellulose membrane as highly porous material with pore sizes from several nanometers to micrometers [2]. However, the absence of pores renders the membrane used in the present study cell-occlusive, suggesting that it will prevent cellular ingrowth from the adjacent connective tissue. In a study utilizing three different expanded polytetrafluoroethylene membrane qualities with different porosities placed on denuded rat calvaria, it was observed that there was a porosity range within which osteogenesis beneath the membrane is optimal, and the material with the smallest internodal distance did not integrate well with the surrounding soft tissue [27]. Thus, the production of a microbial cellulose membrane with different pore sizes will be important according to its application.

Microbial cellulose membrane has been used as a scaffold to substances in order to augment its therapeutic properties [2]. For example, Wan *et al*. [28] developed a microbial cellulose membrane coated with hydroxyapatite, an important compound for bone formation due to its osteoconductivity and bioactivity properties. In the present study, the mesenchymal stem cells aggregated to the cellulose membrane despite the difference of surface texture observed by electron microscopy thus demonstrating its ability to scaffold cells. The maintenance of normal mesenchymal stem cell morphology also suggested biocompatibility of the product. In a study using a cellulose membrane produced by *Acetobacter aceti *the growth of eight types of cells on the membrane was comparable to that obtained in plastics Petri dishes [29]. However, both modification of the ionic charge and adsorption of collagen to membrane were used to promote cellular adhesion. Native and chemically modified bacterial cellulose materials from *A xylinum *was also evaluated as scaffold to chondrocytes suggesting a good potential [30]. On the other hand, tissue culture and full-thickness transcortical bone defects induced in rat's mandible showed better results using expanded polytetrafluoroethylene than alkali-cellulose membrane that showed predominantly endochondral regeneration [16]. According to the authors the alkali-cellulose membrane induced severe inflammatory reaction and appeared to disintegrate.

## Conclusion

The tested microbial cellulose membrane was nonresorbable, induced low inflammatory response, and may be used as a scaffold for stem cells. Further investigations are necessary to confirm its use as barrier material.

## Competing interests

The authors declare that they have no competing interests.

## Authors' contributions

PNM participated in the study design and experimental work. SCR participated in the study design, participated in its coordination and drafted the manuscript. OCMPJ participated in the study design and experimental work, interpreted the results and was responsible for the data analysis and helped to draft the manuscript. VEF performed the histopathological study. SLRL performed the scanning electron microscopy of the biomaterial. JFLM participated in the study design and experimental work. FCLA helped to draft the manuscript. All authors read and approved the final manuscript.
